# *EsDREB2B*, a novel truncated DREB2-type transcription factor in the desert legume *Eremosparton songoricum,* enhances tolerance to multiple abiotic stresses in yeast and transgenic tobacco

**DOI:** 10.1186/1471-2229-14-44

**Published:** 2014-02-10

**Authors:** Xiaoshuang Li, Daoyuan Zhang, Haiyan Li, Yucheng Wang, Yuanming Zhang, Andrew J Wood

**Affiliations:** 1 Key Laboratory of Biogeography and Bioresource in Arid Land, Xinjiang Institute of Ecology and Geography, Chinese Academy of Sciences, Xinjiang Urumqi 830011, China; 2 University of Chinese Academy of Sciences, Beijing 100049, China; 3 Department of Plant Biology, Southern Illinois University, Carbondale, IL 62901-6899, USA

**Keywords:** Cold, DREB, Drought, Heat, Proline, Salt, Stress, Transcript accumulation, Transgenic tobacco

## Abstract

**Background:**

Dehydration-Responsive Element-Binding Protein2 (DREB2) is a transcriptional factor which regulates the expression of several stress-inducible genes. DREB2-type proteins are particularly important in plant responses to drought, salt and heat. DREB2 genes have been identified and characterized in a variety of plants, and DREB2 genes are promising candidate genes for the improvement of stress tolerance in plants. However, little is known about these genes in plants adapted to water-limiting environments.

**Results:**

In this study, we describe the characterization of *EsDREB2B*, a novel *DREB2B* gene identified from the desert plant *Eremosparton songoricum*. Phylogenetic analysis and motif prediction indicate that *EsDREB2B* encodes a truncated DREB2 polypeptide that belongs to a legume-specific DREB2 group. In *E. songoricum*, *EsDREB2B* transcript accumulation was induced by a variety of abiotic stresses, including drought, salinity, cold, heat, heavy metal, mechanical wounding, oxidative stress and exogenous abscisic acid (ABA) treatment. Consistent with the predicted role as a transcription factor*, EsDREB2B* was targeted to the nucleus of onion epidermal cells and exhibited transactivation activity of a GAL4-containing reporter gene in yeast. In transgenic yeast, overexpression of *EsDREB2B* increased tolerance to multiple abiotic stresses. Our findings indicate that *EsDREB2B* can enhance stress tolerance in other plant species. Heterologous expression of *EsDREB2B* in tobacco showed improved tolerance to multiple abiotic stresses, and the transgenic plants exhibited no reduction in foliar growth. We observed that *EsDREB2B* is a functional DREB2-orthologue able to influence the physiological and biochemical response of transgenic tobacco to stress.

**Conclusions:**

Based upon these findings, EsDREB2B encodes an abiotic stress-inducible, transcription factor which confers abiotic stress-tolerance in yeast and transgenic tobacco.

## Background

Abiotic stresses such as drought, salinity, and extreme temperature negatively impact the growth and productivity of crop plants [1,2]. Among these various stresses, drought is the primary factor causing crops to lose productivity [3]. The impacts of drought are likely to become even more pronounced as desertification and global climate change progress around the world. Plants have evolved a diverse set of morphological, biochemical, physiological and molecular adaptations to both survive and sustain growth in harsh environments [4]. Understanding drought response mechanisms is essential to improving drought tolerance and reducing the influence of drought on plant growth and crop yield.

Stress-associated genes and proteins for each of the steps downstream of the drought response can be grouped into two major categories: single functional genes and transcription factors (TFs) [5]. TFs are critical regulators of the changes in gene expression used to respond to environmental stress [6]. A single TF can control the expression of many downstream genes by binding to the cis-acting element in the promoters of the target genes [7]. Current efforts to improve plant stress tolerances by the genetic transformation of TF genes have resulted in several important achievements. The *DREB* (dehydration-responsive element binding proteins) gene family broadly participates in plant stress response pathways [8]. The *DREB2* gene family is particularly important in the responses to dehydration and heat stress [9].

DREB homologs have been identified in a wide variety of plants, including grasses [10-13], fruits [14,15], vegetables [16,17], crops [18-23] and Arabidopsis [24]. Over the past decade, many reports, both in the laboratory and the field, have demonstrated that DREBs hold great potential for improving the stress tolerance of plants in response to drought, salt, cold, and heat [25-28]. Consequently, DREB genes are promising candidates for genetic engineering and have been extensively investigated in recent years. Characterization of *DREB/ERF* genes from wild species (i.e. non-cultivated and non-model plants) may provide novel homologues for breeding stress resistance into crops [9]. Although primarily cloned from herbaceous plants, DREB homologues have been characterized in woody, desert plants such as *Populus euphratica*[29] and *Caragana korshinskii*[30]. Analysis of naturally stress-tolerant plants proffers novel stress-associated gene resources and may enhance our knowledge of DREB expression and function.

*Eremosparton songoricum* (Litv.) Vass. is a leafless perennial dwarf shrub endemic to central Asia [31]. *E. songoricum* is adapted to a harsh desert environment and is a wild pioneer species used to protect the local ecosystem from desertification in the Gurbantunggut desert of Xinjiang. This extremely drought-tolerant leguminous shrub is being developed as a model organism for investigating the morphological, biochemical, physiological and molecular adaptations to a desert environment [32,33]. We report here the cloning of a drought-induced *DREB* gene from *E. songoricum* (*EsDREB2B*). We have characterized EsDREB2B gene expression, subcellular localization, and transactivation activity. In addition, we have investigated the function of *EsDREB2B* in transgenic yeast and tobacco and evaluated transgenic plant tolerance to a suite of abiotic stresses.

## Results

### Characterization of *EsDREB2B* from *E. songoricum*

**Figure 1:**
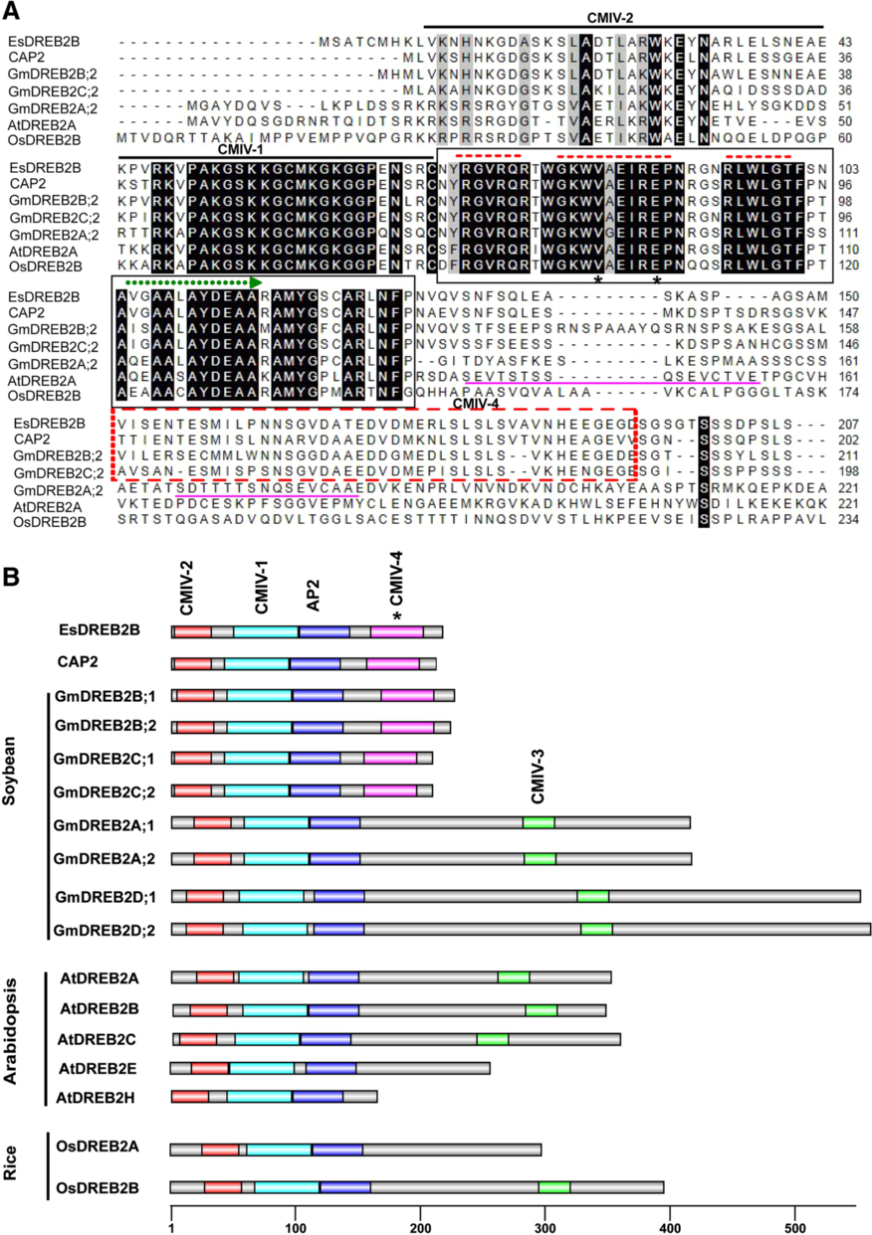
**Alignment and schematic diagram of EsDREB2B and homolog proteins. A** Alignment of the predicted EsDREB2B amino acid sequence with 6 typical DREB2-type proteins from chickpea, soybean, Arabidopsis and rice. Alignment of the predicted polypeptide sequences (residues 1–210) was performed using ClustalX2.1 software. Black and light gray shading indicate identical and conserved amino acid residues. The single lines in black and pink represent predicted motifs (CMIV-1 and CMIV-2) and core negative regulation domain (NRD) of AtDREB2A and GmDREB2A;2 proteins, respectively. The AP2 domain is boxed in black and the three β sheets regions are dash lined in red, the α helix region was labeled in green dash line arrow. Asterisks represent the V14 and E19 conserved amino acids of AP2 domain. The truncated specific CMIV-4 motif was boxed in red. The protein accession numbers are as follows: EsDREB2B (ADV57357.1), CAP2 (ABC49682.1), GmDREB2A;2 (AFU35563.1), GmDREB2B;2 (AAT12423.1), GmDREB2C;2 (AAP83131.1), AtDREB2A (AAL36328.1), OsDREB2B (NP_001055260.2). **B** Architectures of conserved protein motifs. 17 subtype 1 (DREB2-type) transcriptions factors including *E. songoricum*, chickpea, soybean, Arabidopsis and rice are collected for motif detection using MEME online tools. Parameters are as follows: 0 or 1 occurrence of a single motif per sequence, motif width ranges of 6 to 50 amino acids, and 10 as the maximum number of motif. CMIV-1(cyan), CMIV-2 (red) and CMIV-3 (green) motifs are reported by Nakano et al. (2006). The AP2 domain (blue) indicate the conserved AP2/ERF DNA-binding domain, the CMIV-4 conserved motif (pink and marked with*) indicate a specific motif shared with truncated DREB2-type proteins. The domains are drawn to scale. AtDREB2A (AAL36328.1), AtDREB2B (AAP37710.1), AtDREB2C (NP_565929.1), AtDREB2E (NP_181368.1), AtDREB2H (NP_181566.2) are from Arabidopsis; GmDREB2A;1 (AFU35562.1), GmDREB2A;2 (AFU35563.1), GmDREB2B;1 (AAQ57226.1), GmDREB2B;2 (AAT12423.1), GmDREB2C;1 (NP_001236509.1), GmDREB2C;2 (AAP83131.1), GmDREB2D;1 (AFU35564.1), GmDREB2D;2 (AFU35565.1) are from soybean; OsDREB2A (NP_001042107.1) and OsDREB2B (NP_001055260.2) are from rice.

A cDNA clone encoding a DREB2 protein was isolated from two-week-old *E. songoricum* seedlings exposed to 20% (w/v) PEG for 12 h. For clarity and in keeping with the nomenclature established for this gene family in other plants, we have designated the gene *EsDREB2B* and the encoded protein EsDREB2B. *EsDREB2B* [GenBank: HQ687367] was 757 bp in length and contained a single, continuous open reading frame (ORF). The ORF encoded a polypeptide of 207 amino acids with a predicted molecular mass of 22 kDa and a predicted pI of 8.6. The deduced *E. songoricum* polypeptide has significant sequence identity to the conserved AP2/ERF DNA-binding domain [34] and is more than 80% identical to CAP2 from chickpea [21] (Figure 1A). Significant amino acid sequence similarity is also seen with DREB2B deduced polypeptides from soybean (78%), rice (42%) and Arabidopsis (45%). Alignment of the AP2/ERF DNA-binding domain demonstrates that *EsDREB2B* contains the conserved valine (V14) and glutamic acid (E19) residues observed in other DREB predicted polypeptides (Figure 1A) [35,36]. Comparison of the pairwise alignments was conducted using ClustalX 2.1. To determine the architecture of the *EsDREB2B* gene, *E. songoricum* g DNA was amplified using cDNA-specific primers. *EsDREB2B* is an intron-less gene similar to *DREB* genes reported for *Salicornia brachiate* (*SbDREB2A*) [37], chickpea (*CAP2*) [21] and soybean (*GmDREB2B*and *GmDREB2C*) [35].

**Figure 2:**
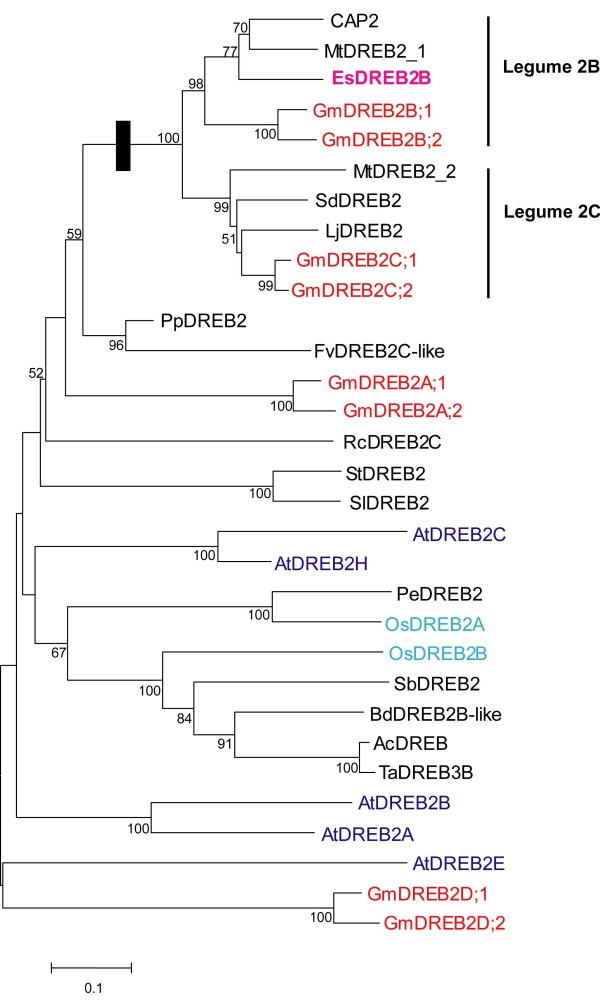
**Phylogenetic analysis of EsDREB2B and homologous proteins.** Phylogenetic tree of subtype1 (DREB2-type) transcription factors in *E. songoricum* (EsDREB2B, pink and bold), soybean (red), Arabidopsis (blue), rice (cyan) and other deduced polypeptides with high sequence identities with EsDREB2B. The neighbor-joining tree is based on an alignment of the complete deduced polypeptide sequences (residues 1–210). Bootstrap values from 1000 replicates were used to assess the robustness of the tree. Solid box (■) indicated truncated sequences clade with CMIV-4 motif. Bootstrap values >50 are shown. **EsDREB2B** (ADV57357.1) is from *E. songoricum*; CAP2 (ABC49682.1) is from chickpea; AtDREB2A (AAL36328.1), AtDREB2B (AAP37710.1), AtDREB2C (NP_565929.1), AtDREB2E (NP_181368.1), AtDREB2H (NP_181566.2) are from Arabidopsis; GmDREB2A;1 (AFU35562.1), GmDREB2A;2 (AFU35563.1), GmDREB2B;1 (AAQ57226.1), GmDREB2B;2 (AAT12423.1), GmDREB2C;1 (NP_001236509.1), GmDREB2C;2 (AAP83131.1), GmDREB2D;1 (AFU35564.1), GmDREB2D;2 (AFU35565.1) are from soybean; OsDREB2A (NP_001042107.1) and OsDREB2B (NP_001055260.2) are from rice; in addition, other protein accession numbers are as follows: **EsDREB2B** (ADV57357.1); MtDREB2B (AFK38091.1); CAP2 (ABC49682.1); MtDREB2C (XP_003597059.1); LjDREB2C (AFK47592.1); SdDREB2C (AFP89590.1); PpDREB2C-like (EMJ17611.1); FvDREB2C-like (XP_004293490.1); StDREB (AEI98833.1); SlDREB (NP_001234689.1); RcDREB2C (XP_002520794.1); PeDREB2 (ABY19375.1); SbDREB2 (AFI71292.1); BdDREB2B-like (XP_003568655.1); AcDREB (ACX94337.1); TaDREB3B (AAX13278.1). Blue = A. thaliana, Red = G. max, Turqoise = O. sativum and Purple = E. songoricum.

EsDREB2B is a member of the AP2/ERF protein family and belongs to the “Group IV, A-2” sub-group [34]. Other members of this sub-group include CAP2 [21], GmDREB2B [35], OsDREB2B [38] and AtDREB2A [24]. Deduced polypeptides of this sub-group each contain single copy of the motifs CMIV-1, CMIV-2 and AP2, and may contain a single copy of CMIV-3 (Figure 1B). CMIV-1 is 27 amino acid residues and defined by the conserved amino acid motif [K/R]GKGGPxN. CMIV-2 is 29 amino acids residues, and CMIV-3 is 25 amino acids residues. CMIV-1 and CMIV-2 are predicted to contain a nuclear localization sequence (NLS) [24,39,40]. EsDREB2B contains a newly identified motif CMIV-4 (Additional file 1: Figure S1). The entire predicted polypeptide EsDREB2B was aligned and compared to 30 DREB2 sequences including AtDREB2A and OsDREB2A (Figure 2). Phylogenetic analysis shows a well-supported group of DREB2 sequences derived from leguminous plants (Fabaceae) including EsDREB2B, CAP2 and GmDREB2B. Additionally, the Fabaceae group contains two well-supported subgroups designated “Legume 2B” and “Legume 2C”. Similar gene tree architectures were obtained using only the AP2 domain, and alternate analytical models for tree construction (Additional file 2: Figure S2).

These legume-specific deduced polypeptide sequences contain canonical DREB2 motifs including the AP2 domain and the CMIV-1 and CMIV-2 motifs [22,34,38]; however, the legume-specific ORFs are truncated (i.e. less than 220 amino acid residues) relative to sequences derived from soybean [35], rice [38] and Arabidopsis [24], lack CMIV-3 and contain a novel motif CMIV-4 (Figure 1). CMIV-4 is 45 amino acid residues and defined by the conserved amino acid motif NSG[V/G]DAX[E/D]D[V/D][D/G]MXX[L/T]SLSL[S/T]V. CMIV-4 is present in each of the truncated DREB2 sequences from legumes, and no other deduced polypeptide sequences in the databases share significant homology with this motif. EsDREB2B and related sequences do not contain a negative regulatory domain (NRD) [22,38,40]. The NRD is present in AtDREB2A, AtDREB2B and AtDREB2C and GmDREB2A;2 [22], and is located immediately downstream of the AP2 domain. Deletion of the NRD in AtDREB2A transforms the protein to a constitutively active form [39].

### Analysis of *EsDREB2B* expression by qRT-PCR in *E. songoricum* seedlings

**Figure 3:**
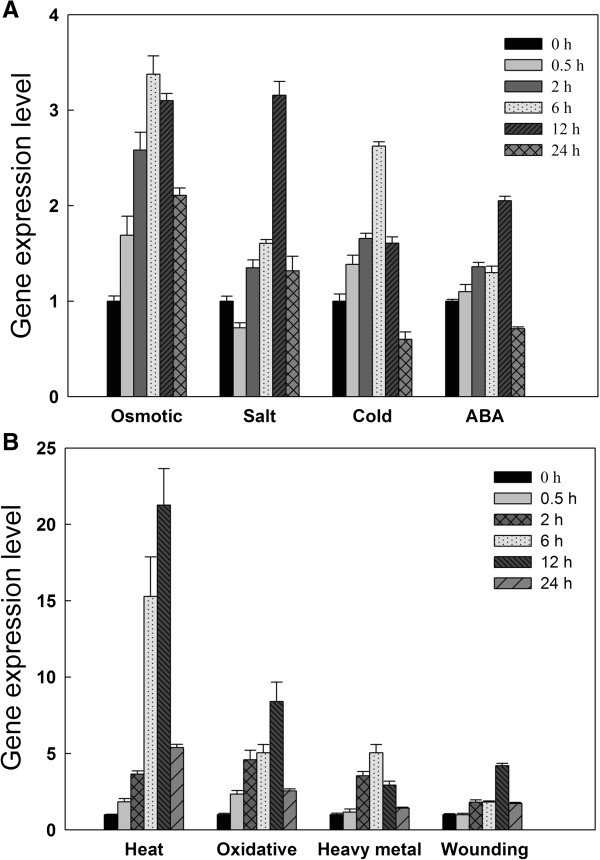
**Expression patterns of *EsDREB2B *gene in response to various stress treatments.***EsDREB2B* expression in two-week old *E. songoricum* seedlings with **A** osmotic, salt, cold stresses and ABA treatment; **B** heat, oxidative, wounding and heavy metal stresses; The relative gene expression level of *EsDREB2B* gene were calculated relative to 0 h under corresponding stress treatment using 2^-ΔΔCT^ method. Error bars indicate SD (n = 3).

Quantitative RT-PCR (qRT-PCR) was employed to analyze the steady-state transcript accumulation of *EsDREB2B* in *E. songoricum* seedlings (Figure 3). *EsDREB2B* transcript was detectable in roots, stem and leaves of unstressed (i.e. control) seedlings, and was most abundant in leaves (Additional file 3: Figure S3). *EsDREB2B* transcript abundance was measured in plants exposed to osmotic stress (20% PEG), salinity (250 mM NaCl), low temperature (4°C), exogenous ABA (100 μM ABA), elevated temperature (42°C), oxidative stress (50 mM H_2_O_2_) and heavy metal (0.5 mM CuSO_4_) (Figure 3). In response to each treatment, *EsDREB2B* steady-state transcript amounts increased, reached a peak at either 6 h or 12 h and subsequently declined at 24 h (Figure 3). With the exception of exogenous ABA exposure and cold stress, *EsDREB2B* transcript was more abundant at 24 h relative to the control (i.e. 0 h). *EsDREB2B* steady-state transcript amounts increased more than 20-fold in response to elevated temperature (Figure 3B). These results suggest that EsDREB2B is widely involved in multiple stress responses, and may function in the ABA-mediated stress tolerance pathway.

### Subcellular localization and transactivation analysis of EsDREB2B protein

**Figure 4:**
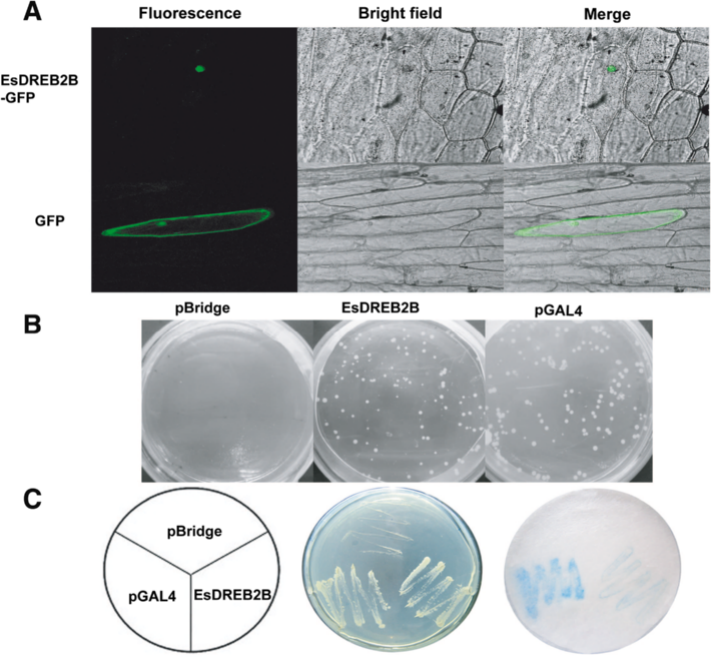
**Subcellular localization and transactivation analysis of EsDREB2B protein. A** GFP and EsDREB2B: GFP fusion protein were transiently expressed under the control of the CaMV 35S promoter in onion epidermal cells and observed with a laser scanning confocal microscope. The images were presented fluorescence, bright field and merge of bright field and fluorescence. **B** The transcriptional activation ability of EsDREB2B was examined using the yeast one-hybrid assay. The growth of yeast cells on SD/-His-Trp medium and **C** β-galactosidase activity was analyzed. PBridge-BD vector served as the negative control and pBridge-BD-GAL4 served as the positive control.

The AP2/ERF family members function as transcription factors in plants and have been documented to localize to the nucleus of Arabidopsis protoplasts [22] and onion epidermal cells [12,41]. The subcellular location of the EsDREB2B protein was analyzed by the transient expression of a GFP fusion protein introduced into onion epidermal cells (Figure 4A). The control protein (p35S-GFP) was distributed in both the cytoplasm and the nucleus of the onion cells, while the EsDREB2B fusion protein (p35S-EsDREB2B: GFP) was only detectable in the nucleus (Figure 4A). The transcriptional activation ability of EsDREB2B was examined using the yeast one-hybrid assay. Yeast cells transfected with the pBridge-BD vector acted as the negative control and were unable to grow on the SD medium without His and Trp (SD/-His-Trp) (Figure 4B). However, the cells harboring pBridge-BD-EsDREB2B or pBridge-BD-GAL4 (the positive control) grew well on the SD/-His-Trp medium (Figure 4B), and these cells turned blue using the *β*-galactosidase activity assay (Figure 4C). These results indicate that EsDREB2B was targeted to the nucleus and exhibited transactivation activity of a GAL4-containing reporter gene. Similar results have been obtained for CAP2 [21] and LcDREB [12].

### EsDREB2B protein expression in transgenic yeast enhances tolerance to multiple stresses

**Figure 5:**
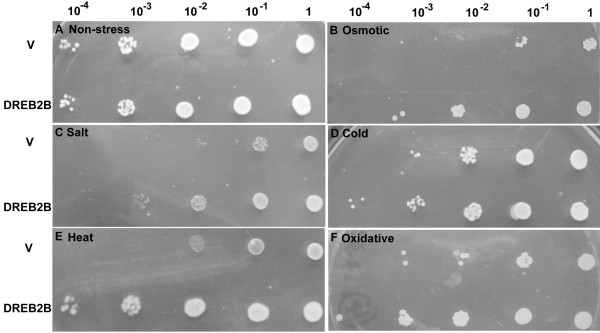
**Growth of *S. cerevisiae *yeast cells transformed with the empty vector (1) PYES2 and with the (2) PYES2-EsDREB2B under different stress conditions.** To test the osmotic and salt tolerances, the same quantity of yeast culture sample was re-suspended in 30% PEG6000 and 5 M NaCl, respectively, at 30°C for 36 h. To test the cold and heat tolerances, the yeast cells were re-suspended in SC-ura medium and placed either in a refrigerator at -20°C alcohol bath or in a water bath at 53°C, respectively, for 24 h. To test oxidative tolerance, yeast cells were incubated in 20 mM H_2_O_2_ at 30°C for 8 h. For non-stress, control (NS), an equivalent number of yeast cells was re-suspended in 200 μl of sterile water and incubated at 30°C for 36 h. Serial dilutions of 1:10 transformed yeast cells were grew on SC-Ura medium for 3 d. **A** non-stress (NS), **B** osmotic stress, **C** salt stress, **D** cold stress, **E** heat stress and **F** oxidative stress.

DREB2 transcriptional factors have not been extensively studied in yeast (*Saccharomyces cerevisiae*). CAP2 has been demonstrated to provide enhanced tolerance to elevated temperature (39°C) in yeast [42]. The *EsDREB2B* cDNA, driven by a galactose-inducible promoter (pYES2), was introduced into *S. cerevisiae* (INVSc1) in order to investigate the ability of EsDREB2B to enhance abiotic stress tolerance (Figure 5). Semi-quantitative RT-PCR analysis confirmed that *EsDREB2B* was expressed in yeast with a peak expression level occurring at 36 h (Additional file 4: Figure S4). Subsequently, all yeast cells were evaluated after 36 h exposure to stress. The growth rate of EsDREB2B-transformed *S. cerevisiae* (pYES2-EsDREB2B) was similar to the growth rate of empty vector controls (pYES2) under non-stress conditions (Figure 5A). *S. cerevisiae* was able to grow in the presence of 30% (w/v) PEG (Figure 5B), 5 M NaCl (Figure 5C), at low temperature (Figure 5D) or elevated temperature (Figure 5E), or in the presence of 20 mM H_2_O_2_ (Figure 5F). EsDREB2B-transformed *S. cerevisiae* had dramatically enhanced growth relative to the control yeast in the presence of PEG (Figure 5B), NaCl (Figure 5C) and heat (Figure 5E). These results indicated that EsDREB2B was functional in yeast cells and improved the tolerance to PEG-, salinity- and heat-stress.

### Evaluation of EsDREB2B in transgenic tobacco in response to drought, salt, cold and heat

**Figure 6:**
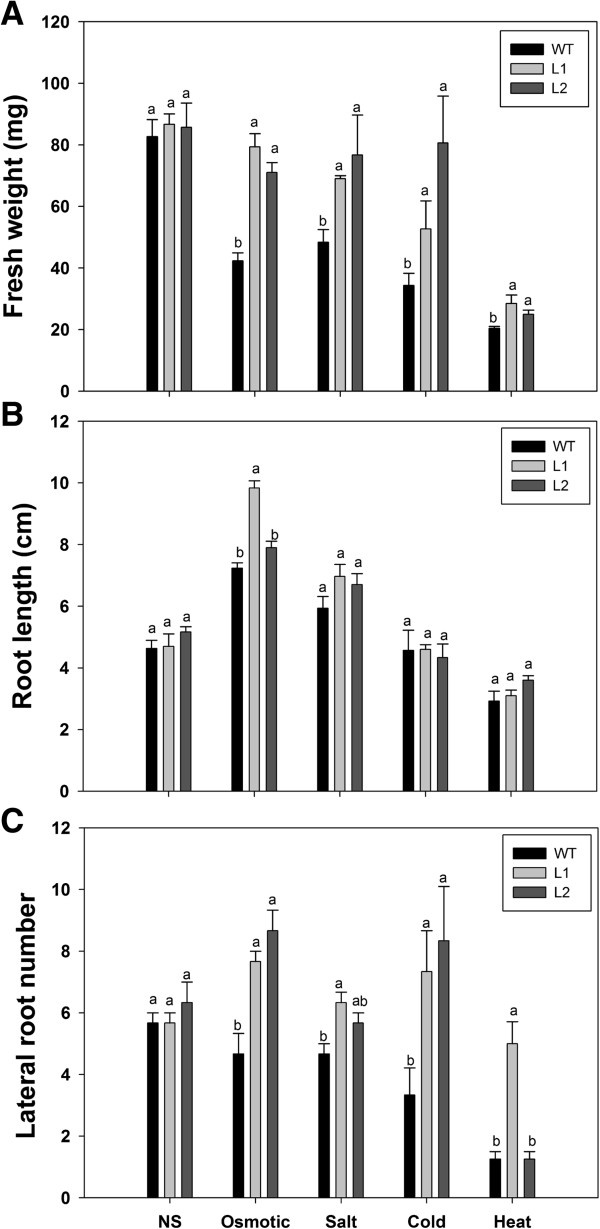
**Fresh weight and root architecture comparison of non-transformed (WT) plants and two *EsDREB2B *transgenic tobacco lines under osmotic, salt, cold and heat stresses.** For osmotic and salt stresses, the two-week old seedlings grown on MS medium transplanted to MS medium supplemented with 200 mM mannitol and 200 mM NaCl for 20 d. For cold and heat stresses, the two-week-old seedlings grown in MS medium were treated for 20 d at 4°C or 7 d at 42°C, respectively. For non-stress control (NS), seedlings were grown in MS culture and kept in normal growth conditions (at 28°C). All plants were grown on vertical MS Plates. **A** fresh weight (expressed in mg), **B** root length (expressed in cm), and **C** lateral root number were determined after treatments. Results are presented means ± SE (n = 24 seedlings). **a–b**: there are no significant differences (p ≥ 0.05) between the lines with the same characters, and significant differences (p < 0.05) between lines with different characters. Significant difference comparison was carried out within stress.

Since *EsDREB2B* transcript accumulation was up-regulated by a number of abiotic stresses (Figure 3) and enhanced the stress tolerance of yeast (Figure 5), transgenic *Nicotiana tobacco* plants containing the EsDREB2B cDNA were generated to evaluate whether overexpression of the ORF could enhance stress tolerance. Hygromycin-resistant transgenic lines were confirmed by PCR and RT-PCR amplification using gene specific primers (data not shown). L1 and L2 were two, randomly selected, independent transgenic lines that overexpressed EsDREB2B. For osmotic- and salinity-stress, two-week-old seedlings were grown on MS medium and transplanted to MS medium supplemented with 200 mM mannitol or 200 mM NaCl for 20 d. For low and elevated temperature-stress, two-week-old seedlings grown in MS medium, transplanted to MS medium and incubated for 20 d at 4°C or 7 d at 42°C, respectively. *EsDREB2B* transgenic tobacco grew normally when un-stressed, and the non-transformed and transgenic lines showed no difference in the fresh weight under non-stress growth conditions (Figure 6A). The fresh weight of WT plants was significantly lower in response to all four treatments (Figure 6A). Transgenic plants maintained significantly higher fresh weight in all four treatments. And with the exception of elevated temperature, the salt and cold-stress L2 transgenic fresh weight values were more similar to unstressed values. Only the osmotic stressed L1 transgenic fresh weight was similar to the unstressed samples. After 20 d of mannitol treatment, dark green leaves with curled edges were observed in both the transgenic and WT plants (Additional file 5: Figure S5). After 20 d of salt treatment, yellow leaves were observed on all of the plants. After 20 d of cold exposure, both WT and transgenic seedlings were slightly wilted. Seedling growth was most affected by elevated temperature. After 7 d of heat exposure, both WT and transgenic lines had pale green cotyledons. Only 17% (i.e. 4/24) of the WT plants survived exposure to elevated temperature, while 92% (44 out of 48) of the transgenic plants (both L1 and L2) survived exposure to elevated temperature.

### Improved root branching in *EsDREB2B* transgenic plants in response to stress

DREB2 proteins such as CAP2 and PgDREB2B have been found to enhanced stress tolerance through regulating root architecture (i.e. root length and lateral root number) [21,43]. We analyzed the root length and lateral root number (Figure 6B, C), and leaf number (Additional file 5: Figure S) of WT and transgenic plants overexpressing EsDREB2B under stress Under non-stress (NS) condition, there were no differences between WT and transgenic plants regarding root length and root number (Figure 6B, C). Root length in L1 transgenic plants was significantly higher in response to osmotic-stress (Figure 6B). With the exception of L2 exposed to elevated temperature, all transgenic plants had significant more lateral roots in response to each of the four treatments (Figure 6C), while leaf number was unchanged (Additional file 5: Figure S5). These results indicated that overexpression of EsDREB2B can enhance tolerance to osmotic-, salt- and temperature-stress.

### *EsDREB2B* transgenic plants were less damaged and accumulated higher levels of proline under stress

**Figure 7:**
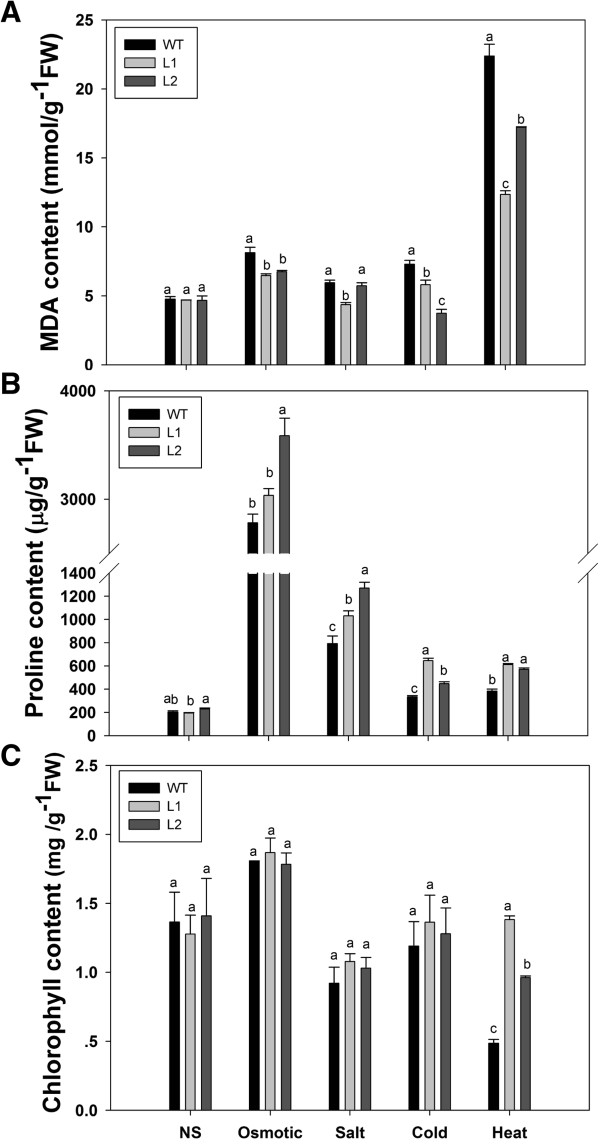
**Comparison of the levels of proline, MDA and chlorophyll between WT and *EsDREB2B*-transformed tobacco after osmotic, salt, cold and heat stresses.** For osmotic and salt stresses, the two-week-old seedlings grown in MS medium under normal conditions (28°C) transplanted to MS medium supplemented with 200 mM NaCl and 200 mM mannitol and stressed for 20 d; for cold and heat stresses, the two-week-old seedlings grown in MS medium and 28°C were treated for 20 d at 4°C and 7 d at 42°C, respectively. Seedlings were kept growing in MS culture and 28°C is the non-stress control (NS). The lethal seedlings were not collected for physiology index measuring. **A** MDA content, **B** proline content, **C** chlorophyll content. Values are mean ± SE from three independent experiments (24 plants of each line were grown for each experiment). **a–c**: there are no significant differences (p ≥ 0.05) between the lines with the same characters, and significant differences (p < 0.05) between lines with different characters. Comparison was carried out within stress.

We monitored several plant stress responses at the physiological level (Figure 7). The MDA, proline and chlorophyll contents did not significantly differ among the WT, L1 and L2 plant lines after 20 d of non-stress (NS) growth (Figure 7A, B, C). In response to all four treatments, L1 transgenic plants had significantly less damage as measured by MDA content when compared with WT plants (Figure 7A). With the exception of salinity-stress, L2 transgenic plants had the same response as L1 transgenic plants. Transgenic plants also accumulated significantly more proline as compared to WT lines under all the stress conditions (Figure 7B). These results indicated that overexpression of EsDREB2B increases the amount of proline in response to osmotic-, salt- and temperature-stress. For chlorophyll content, significant differences between transgenic and WT plants were observed only in response to elevated temperature (Figure 7C).

## Discussion

DRE-binding (DREB) proteins are key components of abiotic stress tolerance in plants [7,8]. DREB2-type transcription factors respond to an array of stressors (i.e. dehydration, salinity, cold, heat and exogenous ABA) and are documented to induce the expression of numerous, downstream, abiotic-stress related genes. Due to their importance, DREB2 genes have been identified in a large number of plant species including soybean [35], rice [38], corn [40], barley [23], chickpea [21] and Arabidopsis [24]. We hypothesize that the identification and characterization of DREB2B homologues from plants adapted dry environments, such as *E. songoricum*, will provide unique insights to DREB function and gene control.

With few notable exceptions (such as *GmDREB2B*), DREB2 genes are induced by both dehydration and heat shock [7,8]. Steady state transcript amounts of *EsDREB2B* do not respond to environmental factors as a typical DREB2B gene. *EsDREB2B* steady state transcript amounts are induced in response to all experimental treatments used in this study: osmotic stress, salinity, low temperature, exogenous ABA, elevated temperature, oxidative stress, heavy metal and wounding. Strong induction in response to dehydration, salinity and elevated temperature coupled with a lack of induction by low temperature is characteristic of *AtDREB2A*, *AtDREB2B*, *OsDREB2A* and *CAP2*[21,24,38]. *EsDREB2B*, like *GmDREB2A*[22], *ZmDREB2A*[40] and *OsDREB2B*[38], exhibited strong induction in response to dehydration, salinity, elevated temperature and low temperature. Interestingly, steady state transcript amounts of the legume-specific, truncated sequences *EsDREB2B*, *CAP2* and *GmDREB2B/C* are induced by exogenous ABA treatment [21,35].

The EsDREB2B deduced polypeptide sequence is a member of the AP2/ERF protein family and most similar to deduced polypeptide sequences from chickpea (CAP2) [21] and soybean (GmDREB2B) [35]. The CMIV-1, CMIV-2 and AP2 domains are found in all previously characterized DREB2 (subtype 1) deduced polypeptides suggesting conserved function of these domains. CMIV-1 and CMIV-2 have been characterized as NLS motifs and AP2 has been determined to act as a DNA-binding domain [24,34,39]. The most intensely studied DREB2 genes (i.e. AtDREB2A/2C, OsDREB2B and ZmDREB2A) contain a CMIV-3 domain in the carboxy-terminal region and the domain has been demonstrated to function as a strong transactivation domain [38-40]. The legume-specific, truncated DREB2 group (i.e. CAP2 and EsDREB2B) contains the CMIV-4 domain. In CAP2, the CMIV-4 domain has been shown to be required for heat tolerance in yeast and may play a critical role in the transactivation of these truncated proteins [21,42]. Shorter DREB2 deduced polypeptides, such as AtDREB2E, AtDREB2H and OsDREB2A lack both the CMIV-3 and CMIV-4 domains and are reported to either be non-responsive to stress or respond to stress without transactivation activity [36,38].

Based upon shared motifs and structural features, we have classified DREB2-type (subtype 1) deduced polypeptides proteins into three groups: 1) non-truncated deduced polypeptides that possess a CMIV-3 domain, 2) deduced polypeptides that lack both the CMIV-3 and CMIV-4 domains and 3) truncated (legume-specific) deduced polypeptides that share a unique CMIV-4 motif. Group 1 DREB2 genes are reported to be significant stress response genes [8,24,36,39] while Group 2 DREB2 genes are not considered to play a key role in stress response [36,38]. These results suggest the possibility that the different groups of DREB2-type proteins with group-specific motifs may have different transactivation mechanisms, and may play different roles in plant stress response.

The ability of DREB2 homologues to enhance osmotic stress tolerance in transgenic plants has been extensively studied [7,8]. Overexpression of AtDREB2A-CA in transgenic Arabidopsis results in greatly enhanced drought- and heat-stress tolerance, slightly enhanced freezing tolerance and no salt-tolerance [39,44]. Similar results have been observed with transgenic Arabidopsis overexpressing OsDREB2B;2 [38], ZmDREB2A [40] and GmDREB2A [22]. Overexpression of CAP2 in transgenic tobacco resulted in enhanced osmotic, salt- and heat-tolerance [21,42]; with the exception of heat-stress tolerance, similar results have been observed with transgenic tobacco overexpressing *PgDREB2A*[43] and *LcDREB*[11]. Overexpression of EsDREB2B in transgenic tobacco was demonstrated to enhance osmotic- and heat-stress tolerance and also enhance cold- and salt-stress tolerance.

Unique among DREB2-type homologues, only *EsDREB2B* and *OsDREB2B* transcript amounts are strongly induced by dehydration, salt, low temperature, elevated temperature and exogenous ABA. However, transgenic Arabidopsis overexpressing OsDREB2B have enhanced tolerance to drought- and heat-stress only. The legume-specific, truncated EsDREB2B transcript is unique among DREB2 homologues in the breadth of transcriptional response and overexpression of the sequence in transgenic tobacco provides enhanced tolerance to osmotic-, salt-, cold- and heat-stress. Further experiments are needed to characterize the mechanisms controlling the accumulation of EsDREB2B transcript and to establish the downstream target promoters potentially regulated by this protein in response to a suite of abiotic stresses.

In DREB2B overexpressing transgenic plants (i.e. Arabidopsis and rice), a common observation is reduced foliar growth as compared to control plants [22,38-40]. In sharp contrast, *EsDREB2B* overexpressing transgenic tobacco demonstrated enhanced growth relative to non-transformed controls under stress conditions. *EsDREB2B* transgenic plants also exhibited no morphological differences as compared to the non-transformed controls. Root architecture and morphology is an important feature of plant growth and development [45]. *EsDREB2B* transgenic tobacco plants had significantly increased numbers of lateral roots. It has been hypothesized that increasing primary root length (e.g. mining for water) and/or increasing lateral root number (e.g. scavenging for water) represent important adaptations to water-limiting environments [46].

The ability to modify essential metabolic processes is key to adapting to adverse environmental conditions. The accumulation of proline is a common response to both biotic and abiotic stress in plants [47] and is postulated to be an adaptive response to declining water potential [48]. Transgenic Arabidopsis and rice plants overexpressing DREB1/CBF have been shown to accumulate proline under control conditions [27]. Several subsequent studies have demonstrated that transgenic plants overexpressing DREB genes will accumulate proline under control conditions and in response to salt-stress [49], osmotic-stress [50], and CuSO4-stress [11]. Overexpression of *EsDREB2B* can influence the physiological and biochemical responses of transgenic tobacco and mediate the accumulation of proline in response to osmotic-, salt-, cold- and heat-stress.

## Conclusions

The cDNA *EsDREB2B* was isolated from the desert plant *E. songoricum. EsDREB2B* encodes a truncated DREB2 deduced polypeptide is a novel truncated DREB2-type protein apparently unique to the Fabaceae. In *E. songoricum*, *EsDREB2B* steady state transcript was detectable under non-stress conditions and accumulated in response to a variety of abiotic stresses. *EsDREB2B* was targeted to the nucleus and exhibited transactivation activity of a GAL4-containing reporter gene. *EsDREB2B* was shown to be a functional DREB protein able to: 1) impart abiotic stress tolerance in transgenic yeast and 2) impart abiotic stress tolerance in transgenic tobacco. In our study, the heterologous expression of EsDREB2B enhanced the tolerance of transgenic tobacco to osmotic-, salt-, cold- and heat-stress. We conclude that *EsDREB2*B is a promising candidate gene for the development of crops with multiple stress tolerances.

## Methods

### Plant material and stress treatments

*Eremosparton songoricum* (Litv) Vass. seeds were collected from the Gurbantunggut Desert, in the Xinjiang Uygur autonomous region of P. R. China (88°24’67”E, 45°58‘11”N, 667 m asl) [32]. Seeds were soaked in 98% (v/v) sulfuric acid for 10–15 minutes to break the physical dormancy of the seeds, washed with ddH_2_O and sown onto moist filter paper. Seedlings were grown in covered petri plates under controlled conditions (25°C, 12 h darkness, 100 mol m^-2^ s^-1^, 60% RH) prior to stress treatment. Plants were monitored daily. The filter paper was never allowed to dry and the plants were not submerged.

*E. songoricum* seedlings were exposed to one of eight stress treatments including PEG6000, salinity, low temperature, elevated temperature, exogenous ABA, H_2_O_2_, exposure to heavy metal and wounding. Two-week-old seedlings were transferred to new petri plates and the filter paper was saturated with 8 mL of one of the following solutions: 20% (w/v) PEG6000, 250 mM NaCl, 100 μM ABA, 50 mM H_2_O_2_, or 0.5 mM CuSO_4_. For the cold and heat treatments, two-week-old seedlings were incubated at 4°C or 42°C, respectively. For mechanical wounding treatment, leaves were cut six times per leaf with a blade [51]. For each time point (i.e. 0.5 h, 2 h, 6 h, 12 h, and 24 h), 3–4 entire plants were harvested, pooled and flash frozen in liquid nitrogen. To serve as a control, two-week-old seedlings were transferred to new petri plates and the filter paper was saturated with 8 mL of water. All plants were maintained under identical growth conditions (i.e. light and RH). To obtain *EsDREB2B*, two-week-old *E. songoricum* seedlings were exposed to osmotic stress (i.e. 20% (w/v) polyethylene glycol (PEG6000)) for 12 h. Harvested samples were flash frozen in liquid nitrogen and stored at -80°C prior to RNA extraction.

### RNA extraction and cDNA synthesis

Total RNA extraction was performed using the RNAiso™ plus reagent (Takara, Japan) following the manufacturer’s instructions with little modification. Genomic DNA contamination was eliminated using RNase-free DNase I (Takara, Japan). The RNA concentration, purity, and integrity were determined using a NanoDrop ND-2000 spectrophotometer (Thermo Fisher Scientific, USA) and were visually assessed via gel electrophoresis (1.2% (w/v) agarose gel). Only RNA samples with a 260/280 ratio of between 1.9 and 2.1 and a 260/230 ratio of higher than 2.0 were used for the subsequent analyses. First-strand cDNA were synthesized from 1 μg of total RNA, l μl of oligo-dT, 1 μl of random six-mers and 4 μl of 5 × Primerscript Buffer (Takara, Japan). The RT-PCR reaction was carried out at 37°C for 30 min on a C1000™ Thermal Cycler (Bio-Rad, USA) at a final volume of 20 μl, and inactivation of the enzyme was completed at 85°C for 5 min. All of the cDNA were stored at -20°C.

### Cloning the *EsDREB2B* cDNA

A conserved partial sequence (ca 300 bp) of the *EsDREB2B* cDNA was amplified from *E. songoricum* cDNA using degenerate primers based upon the AP2 DNA-binding domain of DREB2 deduced polypeptides (G-f: 5’-TGAAACACTGGCAAAATGGAWNGARTAYA-3′ and G-r:5′-CCATACATAGCCATAGCAGCTTCRTCRTANGC-3′) [52]. A full-length *EsDREB2B* cDNA was amplified from *E. songoricum* cDNA using primers derived from the 5′ and 3′-UTR sequences of DREB2 from *Cicer arietinum* (DQ321719) and soybean (AAT1242323) (D-f: 5′-TTCTTCCTCAAATGCCTTCTGG-3′ and D-r: 5′-ATGCAA AACTTACATCAAGACAATG-3′). All primers were designed using Primer Premier 5.0.

### Cloning of a partial *EsDREB2B* gene

DNA was isolated from whole plants of *E. songoricum* using a C-TAB extraction method [53]. To check for the existence of introns within the *EsDREB2B* gene, an ORF-based PCR amplification (primers ORF-f: 5′-ATGAGTGCAACTTGCATGCA-3′ and ORF-r: 5′-AGACAATGAAGGATCGGAGG-3′) was performed using both DNA and cDNA as templates. PCR was performed using *Ex* Taq™ (TaKaRa, Japan), and the reaction conditions were as follows: 30 cycles of denaturation at 94°C for 30 s, annealing at 55°C for 30 s, and extension at 72°C for 45 s. An initial denaturation step of 5 min at 95°C and a final elongation step at 72°C for 10 min were also performed. The final amplification products were checked on a 1.2% (w/v) agarose gel. Amplicons were purified using the EZ Spin Column DNA Gel Extraction Kit (BIO BASIC INC, Canada) and ligated into the pMD™ 19-T vector (according to the manufacturer’s instructions). Positive colonies containing the recombinant plasmids were then sent to the Beijing Genomics Institute (Beijing, China) for DNA sequencing.

### Phylogenetic analysis and conserved protein motif prediction

The resulting DNA sequence and the deduced polypeptide sequences, along with the BLAST algorithm, were used to query the appropriate databases in the National Center for Biotechnology Information (NCBI). Multiple sequence alignment of 31 deduced DREB amino acid sequences was performed using Clustal-W in conjunction with MEGA 5.1 [54]. Phylogenetic trees were generated by the neighbor-joining (NJ) method using MEGA 5.1 (poisson correction and pairwise deletion). Support for nodes on the estimated phylogeny was tested with 1000 bootstrap replicates using the neighbor-joining method [55]. Motif detection was performed with the online tool MEME [56] using the parameters: 0 or 1 occurrence of a single motif per sequence, motif width ranges of 6 to 50 amino acids, and 10 as the maximum number of motif. Motif alignment was performed using MAST (motif alignment and search tool) using default parameters [57].

### Gene expression analysis by qRT-PCR in response to abiotic stress

RNA was isolated from two-week old seedlings exposed to one of eight stress treatments at the time points indicated (see above). To test differential tissue expression, samples of the roots, stems and leaves from two- week-old seedlings grown in normal conditions were collected separately. All cDNA samples were diluted 5-fold with RNase-free water before being used as the template for quantity analysis. QRT-PCR primers were designed outside of the AP2 domain (Dq-f: 5′-AGCCGAGAAGCCTGTTA-3′ and Dq-r: 5′- CCCAAGTCCTTTGCCTA -3). QRT-PCR reactions were performed using SYBR *Premix Ex Taq*^*TM*^ (Takara, Japan) and the CFX96 Real-Time PCR Detection System (Bio-Rad, USA). The reaction mixture consisted of 2 μl of the diluted cDNA samples, 0.4 μl each of the forward and reverse primers (10 μM), 10 μl of the real-time master mix and 7.2 μl of the PCR-grade water for a final volume of 20 μl. Two biology replicates were performed for all of the samples, and three technical replicates of each of the biological replicates with a no-template control (NTC) were made. QRT-PCR protocol was as follows: 30 s of initial denaturation at 95°C, 40 cycles at 94°C for 5 s, and annealing at 60°C for 30 s. The relative expression levels of the *EsDREB2B* gene were calculated relative to the control (at 0 h). Selection of *E. songoricum* reference genes is based upon our previous research [32]. The combination of *EsEF* and *Esα-TUB* are sufficient to reliably normalize the qRT-PCR data in various abiotic stress conditions, and *EsGAPDH* and *Esβ-TUB2* are suitable for different tissue samples.

### Subcellular localization analysis

To determine the subcellular localization of the gene products, the full-length ORF of *EsDREB2B* (without the termination codon) was amplified using the specific primers sub-f and sub-r (sub-f: 5′-ATGAGTGCAACTTGCATGC-3′ and sub-r: 5′-AGACAATGAAGGATCGGAGG-3′). The ORF was fused to the 5’ end of the GFP coding sequence and sub-cloned to pBI221 under control of the CaMV 35S promoter. The fusion construct (p35S-EsDREB2B: GFP) and the control (p35S-GFP) were transformed into onion epidermis cells by particle bombardment (Bio-Rad, Hercule, CA). Green fluorescence was observed under a confocal microscope (Olympus FV500, Olympus, Japan).

### Trans-activation assay using yeast one-hybrid system

The entire ORF of *EsDREB2B* was amplified with specific primers (Y-f and Y-r), and the primer sequences were Y-f: 5′-GTCGACTGATGAGTGCAACTTGCATGC-3′ and Y-r: 5′-CTGCAGTCAAGACAATGAAGGATCGG-3′). The amplification product was inserted between the *Pst*I and *Sal*I restriction sites of the yeast expression vector pBridge, which also contained the binding domain (BD) of GAL4 (pBridge -BD-EsDREB2B). The recombinant plasmids were introduced into *Saccharomyces cerevisiae* (strain AH109 yeast strain carrying the His3 and LacZ reporter genes) per the manufacturer’s instructions (Clontech, USA). Yeast cells containing only GAL4 (pBridge-BD-GAL4) or the pBridge vector (pBridge-BD) were used as positive and negative controls, respectively. Successful transformants were selected by growth on SD media without His and Trp at 30°C for 3 days. Positive colonies were confirmed by yeast colony PCR. In addition, the *β-*galactosidase activity of the transformant colonies was examined by incubating the colonies in Z buffer with x-gal at 30°C.

### *EsDREB2B* stress tolerance studies in yeast

The ORF of *EsDREB2B* was amplified from pMD19-T-*EsDREB2B* using the primer pair YE-f and YE-r and contained the sequences for the *Bam*HI and *Kpn*I restriction enzymes sites. The primer sequences were: YE-f: 5′-GGTACCATGAGTGCAACTTGCATGCA-3′and YE-r: 5′-CGGATCCGTCAAGACAATGAAGGATCGG-3′. The ORF was inserted into the *Bam*HI and *Kpn*I sites of the yeast expression vector pYES2 (Invitrogen, USA), which contains a URA3 selection marker driven by the GAL1 promoter. Subsequently, the pYES2-EsDREB2B plasmid and the empty pYES2 control plasmids were introduced into yeast strain INVSc1 (Invitrogen, USA) using a lithium acetate procedure, according to the pYES2 vector kit instructions (Invitrogen, USA). The transformants were screened by growth on a uracil-deficient synthetic complete (SC-ura) medium with 2% (w/v) glucose at 30°C. Positive colonies were verified by plasmid PCR and double digestion (with *BamH*I and *Kpn* I) analysis.

To investigate the expression of *EsDREB2B* in the transgenic yeast, total RNA from yeast cells harboring pYES2-EsDREB2B were isolated using the TRIzol Reagent (Invitrogen, USA). A semi-quantitative RT-PCR assay was performed using the YE-f and YE-r primer pair for the target gene, with ACT1 as the reference gene; the ACT1 primer set conforms to that of Teste et al. [58]. For the stress assay, yeast cells harboring both pYES2-EsDREB2B and the empty pYES2 vector (control) were incubated in SC-ura liquid medium containing 2% (w/v) glucose at 30°C for approximately 20 h. The densities of the yeast cell cultures were measured (by OD600) after incubation. The culture samples were adjusted to contain an equal number of cells (OD600 = 0.4) in 10 ml of induction medium (SC-ura medium supplemented with 2% w/v galactose) and cultivated for 36 h to promote the expression of the *EsDREB2B* gene. After incubation, the yeast cell densities were recalculated and again adjusted to contain an equal number of cells (OD600 = 1) in 200 μl solutions with different chemicals for the abotic stress assays. For the drought and salt stresses, the same quantity of culture sample was resuspended in 30% (w/v) PEG6000 or 5 M NaCl, respectively, at 30°C for 36 h. For the cold and heat stresses, yeast cells were resuspended in SC-ura medium and either incubated in a -20°C alcohol bath for 24 h or in a water bath at 53°C for 24 h [59]. For oxidative stress, yeast cells were incubated in 20 mM H_2_O_2_ at 30°C for 8 h. For the control, an equivalent number of yeast cells was resuspended in 200 μl of sterile water and incubated at 30°C for 36 h. After the stress treatments, the cells were 10-fold serially diluted (1, 10^-1^, 10^-2^, 10^-3^, and 10^-4^), and 2 μl aliquots of each dilution were spread on SC-ura medium (supplemented with 2% w/v glucose) and incubated at 30°C for 3 days to observe their growth performance. Each experiment was carried out in triplicate.

### The generation of transgenic tobacco plants

To verify the stress tolerance function of the *EsDREB2B* cDNA, complete ORF was inserted in the plant expression vector pCAMBIA 1301, which was digested by *Pst*I and *Kpn*I. The pCAMBIA 1301-*EsDREB2B* vector was introduced into tobacco (Petite Havana SR1) by *Agrobacterium tumefaciens*-mediated leaf dish transformation (LBA4404) [60]. The transgenic nature of the tobacco was confirmed by a hygromycin screen (50 μg/ml) as well as PCR and RT-PCR amplification using the *EsDREB2B* gene specific primer set ORF-f and ORF-r (see above).

### Evaluation of transgenic tobacco plants for abiotic stress tolerance

Two- week-old homozygous T2 transgenic tobacco seedlings were used in various stress tolerance experiments. The WT line and two randomly selected transgenic lines designated L1 and L2 were used for further analysis. Seeds were sown onto solid MS medium plates and allowed to germinate and grow in a 28°C/25°C growth chamber under 8 h darkness and 60% RH. For subsequent treatment all seedlings (including controls) were transplanted to solid MS medium. For osmotic stress and salinity, two-week-old seedlings were transplanted to solid MS medium supplemented with either 200 mM mannitol or 200 mM NaCl for 20 d and grown vertically. For the low temperature and elevated temperature, two-week-old seedlings were transplanted to solid MS medium maintained for 20 d at 4°C or 7 d at 42°C, respectively and grown vertically. For a control, seedlings were grown vertically in MS culture and kept in normal growth conditions (at 28°C). After the treatment, plant growth parameters such as root length, lateral root number, leaf number, and fresh weight were monitored. The length of the main root was measured with a ruler. Physiological indexes, including the amounts of proline, methane dicarboxylic aldehyde (MDA) and chlorophyll, were measured after the stress treatments according to the methods described [61-63]. The plant’s phenotype was photographed before and after the stress treatment.

### Statistical analysis

Statistical analyses were performed using SPSS software (standard version 11.5 released for Windows, SPSS Inc., IL, USA). All data were analyzed using a one-way analysis of variance (ANOVA) at the 95% confidence level. An LSD multiple comparison test was then used to compare the significant differences. The data shown are the mean values ± SE of at least three replicates, and the significance level relative to controls is P < 0.05. The figures were generated using Sigmaplot10.0. The image-processing software used was Adobe Illustrator CS.

## Abbreviations

TF: Transcription factors; DREB: Dehydration-responsive element binding protein; ORF: Open reading frame; NJ: Neighbor-joining; PEG: Polyethylene glycol; qRT-PCR: Quantitative RT-PCR; NTC: No-template control; ABA: Exogenous abscisic acid; NLS: Nuclear localization sequence; NRD: negative regulatory domain; MDA: Methane dicarboxylic aldehyde.

## Competing interests

The authors declare that they have no competing interests.

## Authors’ contribution

XSL performed the gene cloning, sub-cellular location and transgenic tobacco assays, and wrote the manuscript. DYZ designed, supervised and financed this work, and assisting with editing the manuscript. HYL carried out the gene expression and transgenic yeast experiments. YCW provided advice on the yeast activation assay, transgenic yeast assay and assisted with data analysis. YMZ designed and prepared the manuscript. AJW assisted with data analysis and edited the manuscript. All authors read and approved the final manuscript.

## Supplementary Material

Additional file 1: Figure S1 Sequence logo for the CMIV-4 motif of the truncated proteins. Click here for file

Additional file 2: Figure S2 Phylogenetic tree of EsDREB2B and homolog proteins. *E. songoricum* (EsDREB2B, pink and bold), soybean (red), Arabidopsis (blue), rice (cyan) and other proteins with high sequence identities with EsDREB2B. The neighbor-joining tree is based on the Jone-Taylor-Thornton model (pairwise deletion) with an alignment of the complete protein sequence. Bootstrap values from 1000 replicates were used to assess the robustness of the tree. Bootstrap values >50 are shown. Click here for file

Additional file 3: Figure S3 Tissue expression pattern of *EsDREB2B* in two-week old *E. songoricum* seedling under normal condition. The relative gene expression level of *EsDREB2B* gene were calculated relative to leaf sample using 2^-ΔΔCT^ method. Error bars indicate SD (n = 3). Click here for file

Additional file 4: Figure S4 Semi-quantitative RT-PCR analysis of induced gene expression pattern of *EsDREB2B* in recombinant yeast. The suppressive and galactose-induced expression of control yeast (pYES2) for 36 h (Lanes 1, 2). The suppressive expression of recombinant yeast (*pYES2-EsDREB2B*) for 36 h (lane3) and galactose-induced expression of recombinant yeast for 12 h (lane 4), 24 h (lane 5), 36 h (lane 6), 48 h (lane 7) and 60 h (lane 8). Click here for file

Additional file 5: Figure S5 Phenotype and leaf number comparison of non-transformed (WT) plants and two *EsDREB2B* transgenic tobacco lines under osmotic, salt, cold and heat stresses. A The photograph was taken beforeand after stress; B leaf number were counted after stress treatments. Results are presented means ± SE (n = 24 seedlings). Significant difference comparison was carried out within stress. Click here for file
