# Supplementary figures and images for: EsDREB2B, a novel truncated DREB2-type transcription factor in the desert legume Eremosparton songoricum, enhances tolerance to multiple abiotic stresses in yeast and transgenic tobacco
Source: BMC Plant Biol. 2014 Feb 10;14:44. doi: 10.1186/1471-2229-14-44 (PMC3940028; doi:10.1186/1471-2229-14-44)

**Additional file 1:** Sequence logo for the CMIV-4 motif of the truncated proteins.

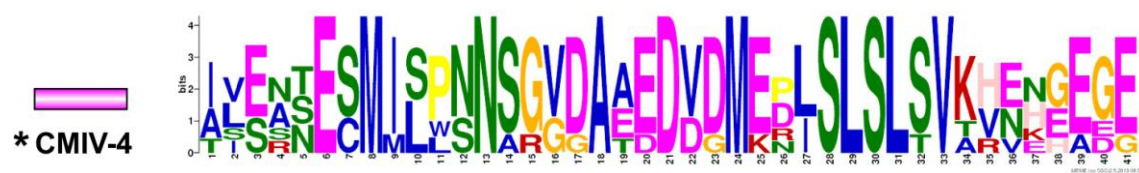

Supplement: Additional file 1: Figure S1 — Sequence logo for the CMIV-4 motif of the truncated proteins. [file 1471-2229-14-44-S1.pdf]

**Additional file 2:** Phylogenetic tree of EsDREB2B and homolog proteins.

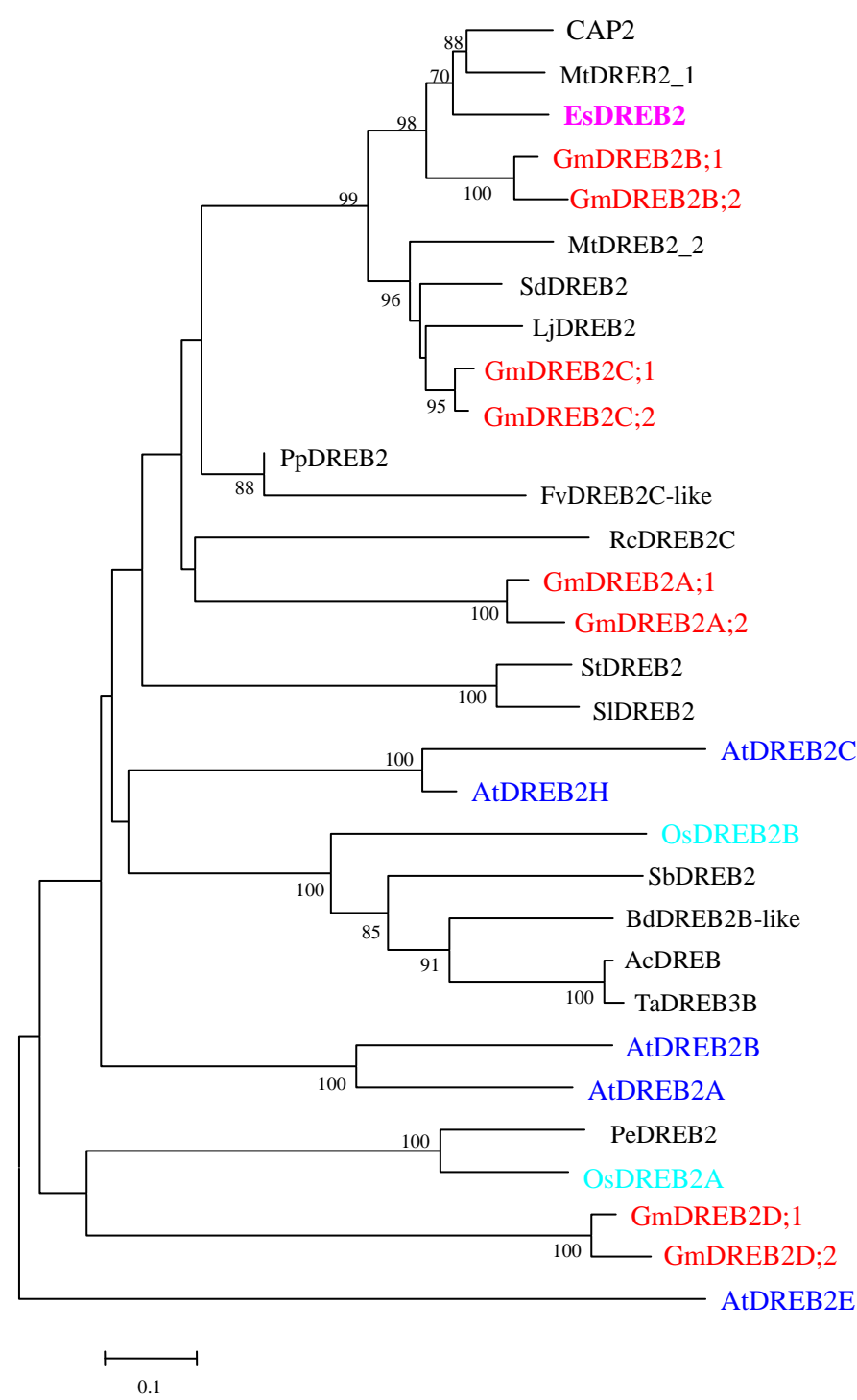

Supplement: Additional file 2: Figure S2 — Phylogenetic tree of EsDREB2B and homolog proteins. E. songoricum (EsDREB2B, pink and bold), soybean (red), Arabidopsis (blue), rice (cyan) and other proteins with high sequence identities with EsDREB2B. The neighbor-joining tree is based on the Jone-Taylor-Thornton model (pairwise deletion) with an alignment of the complete protein sequence. Bootstrap values from 1000 replicates were used to assess the robustness of the tree. Bootstrap values >50 are shown. [file 1471-2229-14-44-S2.pdf]

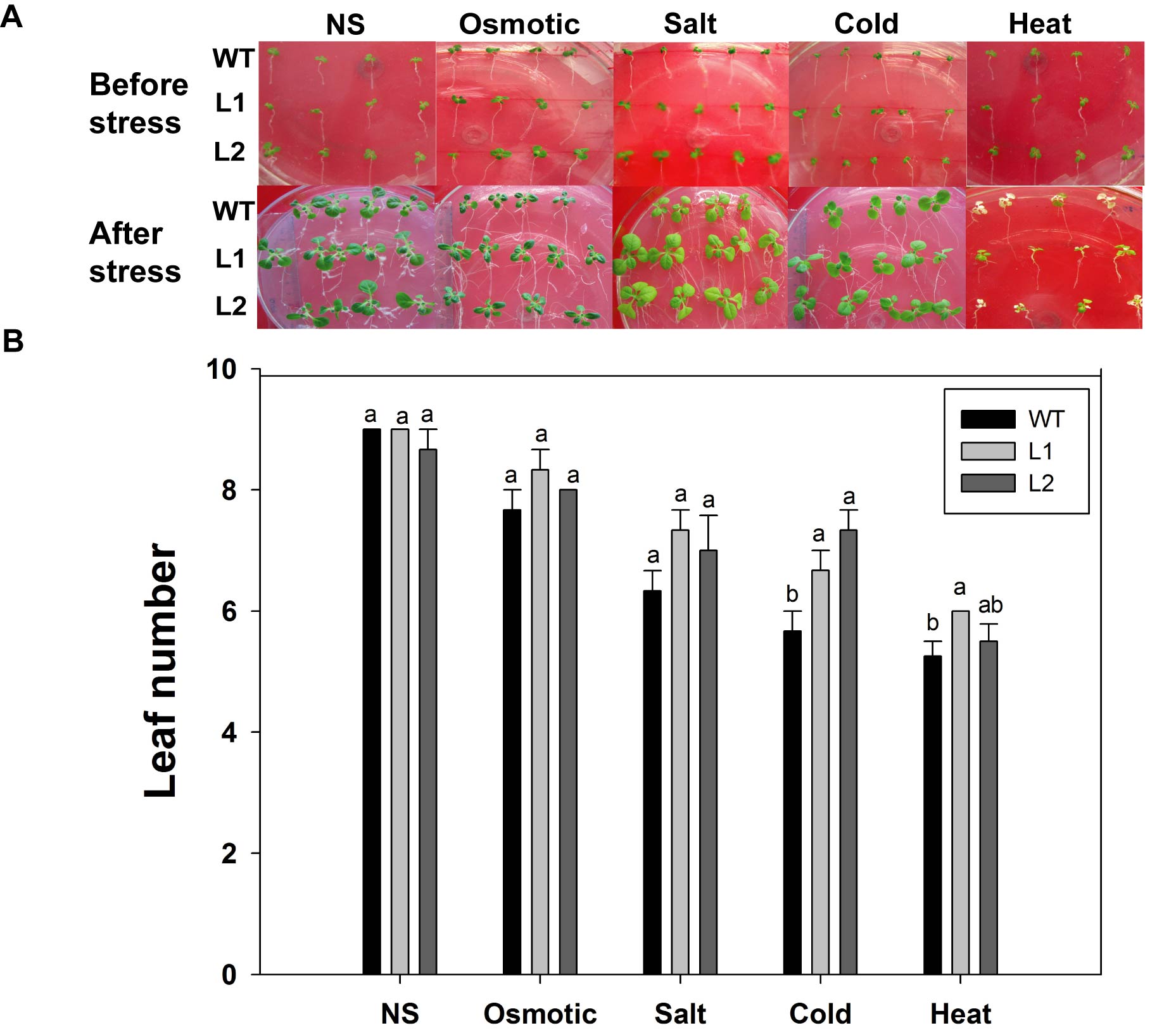

Supplement: Additional file 5: Figure S5 — Phenotype and leaf number comparison of non-transformed (WT) plants and two EsDREB2B transgenic tobacco lines under osmotic, salt, cold and heat stresses. A The photograph was taken beforeand after stress; B leaf number were counted after stress treatments. Results are presented means ± SE (n = 24 seedlings). Significant difference comparison was carried out within stress. [file 1471-2229-14-44-S5.jpeg]
