# Supplementary material for: EsDREB2B, a novel truncated DREB2-type transcription factor in the desert legume Eremosparton songoricum, enhances tolerance to multiple abiotic stresses in yeast and transgenic tobacco
Source: BMC Plant Biol. 2014 Feb 10;14:44. doi: 10.1186/1471-2229-14-44 (PMC3940028; doi:10.1186/1471-2229-14-44)

Additional file 3:

Tissue expression pattern of *EsDREB2B* in two-week old *E. songoricum* seedling under normal condition. The relative gene expression level of *EsDREB2B* gene were calculated relative to leaf sample using $2^{-\Delta\Delta CT}$ method. Error bars indicate SD (n = 3).

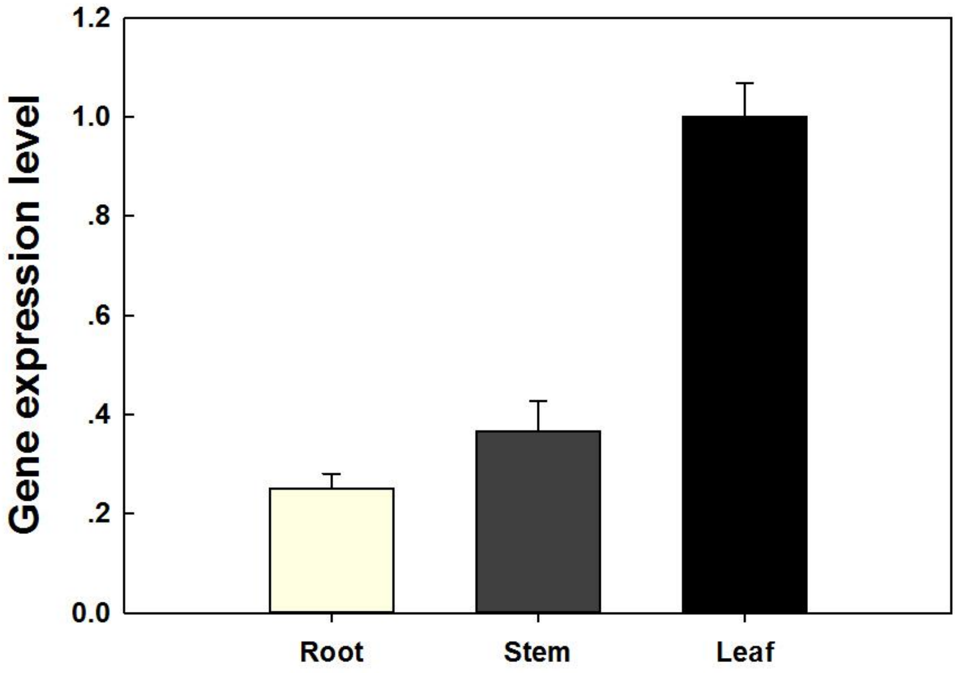

Supplement: Additional file 3: Figure S3 — Tissue expression pattern of EsDREB2B in two-week old E. songoricum seedling under normal condition. The relative gene expression level of EsDREB2B gene were calculated relative to leaf sample using 2-ΔΔCT method. Error bars indicate SD (n = 3). [file 1471-2229-14-44-S3.pdf]
