# Supplementary material for: EsDREB2B, a novel truncated DREB2-type transcription factor in the desert legume Eremosparton songoricum, enhances tolerance to multiple abiotic stresses in yeast and transgenic tobacco
Source: BMC Plant Biol. 2014 Feb 10;14:44. doi: 10.1186/1471-2229-14-44 (PMC3940028; doi:10.1186/1471-2229-14-44)

Additional file 4: Semi-quantitative RT-PCR analysis of induced gene expression pattern of *EsDREB2B* in recombinant yeast. The suppressive and galactose -induced expression of control yeast (pYES2) for 36 h (Lanes 1, 2). The suppressive expression of recombinant yeast (*pYES2-EsDREB2B*) for 36 h (lane3) and galactose-induced expression of recombinant yeast for 12 h (lane 4), 24 h (lane 5), 36 h (lane 6), 48 h (lane 7) and 60 h (lane 8).

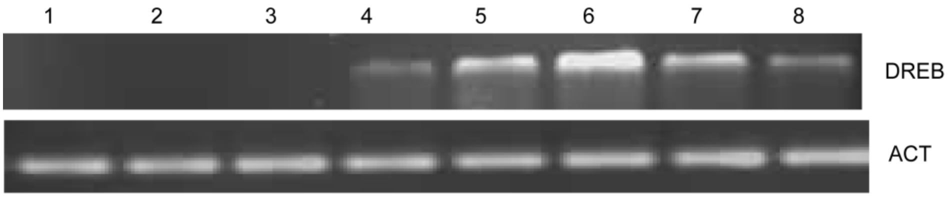

Supplement: Additional file 4: Figure S4 — Semi-quantitative RT-PCR analysis of induced gene expression pattern of EsDREB2B in recombinant yeast. The suppressive and galactose-induced expression of control yeast (pYES2) for 36 h (Lanes 1, 2). The suppressive expression of recombinant yeast (pYES2-EsDREB2B) for 36 h (lane3) and galactose-induced expression of recombinant yeast for 12 h (lane 4), 24 h (lane 5), 36 h (lane 6), 48 h (lane 7) and 60 h (lane 8). [file 1471-2229-14-44-S4.pdf]
